# Registered Nurses’ Perceptions about the Situation of Family Caregivers to Patients with Heart Failure - A Focus Group Interview Study

**DOI:** 10.1371/journal.pone.0160302

**Published:** 2016-08-09

**Authors:** Annelie K. Gusdal, Karin Josefsson, Eva Thors Adolfsson, Lene Martin

**Affiliations:** 1 School of Health, Care and Social Welfare, Mälardalen University, Eskilstuna, Sweden; 2 Faculty of Caring Science, Work Life and Social Welfare, University of Borås, Borås, Sweden; 3 Centre for Clinical Research, Uppsala University, County Council of Västmanland, Västerås, Sweden; 4 Department of Primary Health Care, Västmanland County Hospital, Västerås, Sweden; 5 School of Health Sciences, City University, London, United Kingdom; University of Florence, ITALY

## Abstract

**Introduction:**

Heart failure is a growing public health problem associated with poor quality of life and significant morbidity and mortality. The majority of heart failure care is provided by family caregivers, and is associated with caregiver burden and reduced quality of life. Research emphasizes that future nursing interventions should recognize the importance of involving family caregivers to achieve optimal outcomes.

**Aims:**

The aims of this study are to explore registered nurses’ perceptions about the situation of family caregivers to patients with heart failure, and registered nurses’ interventions, in order to improve family caregivers’ situation.

**Methods:**

The study has a qualitative design with an inductive approach. Six focus group interviews were held with 23 registered nurses in three hospitals and three primary health care centres. Data were analysed using qualitative content analysis.

**Results:**

Two content areas were identified by the a priori study aims. Four categories and nine sub-categories emerged in the analysis process. The content area “Family caregivers' situation” includes two categories: “To be unburdened” and “To comprehend the heart failure condition and its consequences”. The content area “Interventions to improve family caregivers' situation” includes two categories: “Individualized support and information” and “Bridging contact”.

**Conclusions:**

Registered nurses perceive family caregivers' situation as burdensome, characterized by worry and uncertainty. In the PHCCs, the continuity and security of an RN as a permanent health care contact was considered an important and sustainable intervention to better care for family caregivers' worry and uncertainty. In the nurse-led heart failure clinics in hospitals, registered nurses can provide family caregivers with the opportunity of involvement in their relative's health care and address congruence and relationship quality within the family through the use of "Shared care" and or Family-centred care. Registered nurses consider it necessary to have a coordinated individual care plan as a basis for collaboration between the county council and the municipality.

## Introduction

Heart failure (HF) is a growing public health problem associated with poor quality of life and significant morbidity and mortality [1]. Enhanced pharmacological and invasive therapies have improved prognosis in HF but have also increased the number of patients living with HF. Improved survival after acute coronary syndromes, and population ageing are further contributing to an increased prevalence of HF [1,2]. HF is the single most frequent cause of hospitalization in persons ≥ 65 years [3,4]. Nearly half of persons ≥ 70 years are readmitted to hospital within six months [5], which could be prevented with improved treatment adherence and availability of adequate multidisciplinary professional support [6].

The majority of HF care is provided by family caregivers [7] who have an essential role in their relative's health outcomes and HF self-care [8,9,10,11]. Family caregiving includes assisting with symptom control, supporting patients in complex medical and self-care behaviours, care transitions and decision-making [7,12,13,14]. They provide emotional, physical and cognitive support during the often erratic course of HF with periods of stability, interspersed with exacerbations and unpredictable acute hospitalizations [15,16,17]. Support from family caregivers has a positive impact on patient outcomes and patient’s quality of life, and reduces the incidence of re-hospitalization [8,18,19]. Family caregiving can be rewarding and satisfying [8] but it is also associated with significant caregiver burden, reduced quality of life and depression [7,20,21]. Family caregivers' poor health status might affect patients’ condition and prognosis negatively [22,23]. Most importantly, family caregivers feel that they do not receive sufficient support or recognition from health care professionals [24] and they have expressed a need for increased involvement in the care of their relatives, together with health care professionals [25].

The goal for management of HF is to provide a “seamless” system of care, embracing programs in both primary and hospital health care [2]. The HF programs should be designed to improve outcomes through structured follow-up with patient education, optimization of medical treatment, psychosocial support and improved access to care [26]. The European Society of Cardiology (ESC) guidelines advocate the implementation of nurse-led HF programs to achieve optimal management of HF [2]. Follow-up after hospitalization at a nurse-led HF clinic has been shown to reduce mortality, number of readmissions and days in hospital [27,28,29,30]. Despite good results, nurse-led HF clinics in Sweden are not fully deployed in hospitals and only to a limited extent in primary care, even though the majority of patients with heart failure are managed in primary care [31,32]. Yet, registered nurses (RNs) have a key position in coordinating the overall HF care and the role of the RN is broad [33]. It can involve home visits, telephone contact, facilitating tele-monitoring, running nurse-led HF clinics, as well as providing education for patients, family caregivers and health professionals involved in the HF management [33]. Previous research also emphasizes the central role that RNs have in providing psychosocial support and meeting the needs of family caregivers to patients with HF [23,32,34].

Research on family caregiving in HF is advancing, but to our knowledge the research on family caregivers' situation and needs from the nurses’ perspective is limited. An underlying assumption in this study is that the quality of care for family caregivers is influenced by RNs' perceptions of family caregivers' situation and needs [35]. As RNs may have ambivalent perceptions about family caregivers' situation and their needs, RNs' care for family caregivers and patients may be affected in a negative direction. Wright and Leahey [35] propose that when family caregivers' needs are addressed and acknowledged, the quality of care for both patient and family caregiver is improved. Given the importance of family caregiving in HF and the role RNs can play in supporting family caregivers in their caregiving [35,36], there is a need to explore RNs' perceptions of family caregivers' situation and RNs’ own role in improving family caregivers' situation. Moreover, as most HF nursing interventions primarily focus on patients to improve outpatient self-care [37] increased knowledge of nursing interventions which focus on family caregivers, as well as patients, is needed. Thus, the aims of this study are to explore RNs’ perceptions about the situation of family caregivers to patients with HF living in their own home, and RNs’ interventions to improve family caregivers’ situation.

## Methods

The study has a qualitative design with an inductive approach using focus group interviews (FGIs) for data-collection [38,39,40].

### Study setting, participants and recruitment procedure

The study was conducted in one county in the mid-east of Sweden from June to October 2014. Sweden's 21 county councils and 290 municipalities are the health authorities responsible for planning, financing and operating health care [41]. The county councils have a mandatory commitment to provide healthcare services which are divided into primary, county and regional level. The municipalities have a mandatory commitment to provide health and social care for disabled and persons ≥ 65 years in ordinary homes and nursing homes. In the present county council the responsibility for health care in ordinary homes was shifted from county council to the county's 10 municipalities. The shift is in line with 20 of Sweden's 21 county councils and was carried out two years prior to the present study [41]. In the present county council, all of the hospitals and one of the primary health care centres (PHCCs) had a nurse-led HF clinic, or designated time for follow-up of patients with HF, which is representative for a majority of the county councils in Sweden [42].

Participants of interest for this study were RNs working in both primary and secondary county council care who presumably met patients with HF and their family caregiver on a daily basis. The county has three public hospitals; one county hospital and two rural. All three hospitals were asked, and agreed, to participate in the study. In the county there are 29 PHCCs. Of the 12 PHCCs who met the inclusion criteria, six were randomly selected and asked to participate, and three of these agreed to participate in the study. The inclusion criteria were RNs who a) worked at PHCC with a minimum of 2000 listed patients ≥ 65 years or, b) worked in the medical clinic of a county or rural hospital and, c) had worked at the present unit for a minimum of one year.

The first author contacted the RN in charge in each of the participating health units. Verbal information on the study was given and a time and place for the FGI was agreed upon. Thereafter, written information on the study and a written form of informed consent were sent to the RN in charge, who distributed these to RNs who met the inclusion criteria. In total, 23 RNs were included in the study; 10 RNs from three hospitals and 13 RNs from three PHCCs. Demographic data for the participants are given in Table 1. All RNs met patients with HF, and sometimes their family caregivers, in their daily work.

**Table 1:** Participants in focus group interviews divided by health care organization.

Health care organization	Hospital	Primary health care centre
No. of focus group interviews conducted	3	3
No. of participants, female/male	9/1	13/0
Age in years, median (range)	43 (29-64)	53 (47-63)
Overall professional experience in years, median (range)	15 (2-44)	26 (14-38)
Professional experience in years in present health care unit, median (range)	15 (2-44)	11 (1-34)
No. of participants with designated time for follow-up of patients with HF	4	0
No. of participants with postgraduate education in cardiac care	4	0
No. of participants with specialist nurse education in district care	0	10

### Data collection

With the intent to explore in-depth and wide-ranged experiences and perceptions, FGIs were used. This mode of data collection is shown to elicit rich information through the dynamics and interaction generated within the group [39]. Six FGIs were performed with 3-5 RNs in each group [38,39]. For practical reasons all FGIs were conducted in the RNs’ respective workplaces. RNs in each FGI worked in the same health unit thus an intra-group homogeneity was achieved, shown to improve group dynamics and interaction [40]. All participants completed a questionnaire with their background data before the FGI started. The FGIs were moderated by the first author, a doctoral candidate with experience and training as a moderator of FGIs. A semi-structured interview guide (Table 2) was used with open-ended questions, adding follow-up questions and probes when needed [39,43]. The interview guide was developed by the research group based on the literature on family caregiving and HF [15,25,44]. The moderator balanced the FGIs so that all questions in the interview guide were evoked. An assistant moderator, a doctoral candidate with experience of FGIs, took observational notes and helped to recapture and summarize points of particular relevance to the study aims at the end of each interview. The participants then reflected, verified and further developed the content, a recommended method of validation of FGIs [39]. The interviews lasted 45-60 minutes and were digitally audio recorded. A summary based on observational notes and a debriefing session were performed by the moderator and the assistant moderator immediately after each interview. The interviews were transcribed verbatim in its entirety by the first author and the transcriptions were verified by the co-authors.

**Table 2:** Interview guide.

**Main questions**
1.If you look at an ordinary day, can you tell me about specific situations when you have met patients with heart failure and their family caregivers?
2.What are your perceptions about family caregivers' situation in their everyday life?
3.Do family caregivers in some way express their needs or talk about their everyday life and situation, and if so can you tell me about that?
4.How does family caregiving affect family caregivers’ relationships with their ill relative, their friends, family, personal interests and work?
5.What can health care do to support or improve the situation for family caregivers?
**Follow-up questions and probes for clarification**
1.What do you mean?
2.How did you feel then?
3.What did you think then?
4.… or through confirming what had just been said.

### Data analysis

Qualitative content analysis [45,46] was performed using the QSR NVivo^®^ software program [47]. This method of analysis was chosen as it retains closeness to data from the FGIs and enables categories, which represents participants' perceptions, to emerge in a systematic way [45,46]. All authors have long experience and training in qualitative analysis and the first author has additional training in the use of the NVivo^®^ [47]. Initially, all authors read the interviews several times to get a general sense of the whole, and two content areas were identified by the a priori study aims. Coding and categorization were then carried out inductively in several stages. First, the text within each content area was divided into meaning units, each comprising several words, sentences, or paragraphs containing aspects related to each other through their content and context. Second, taking the context into consideration, the meaning units were condensed and each was labelled with a code. Third, the codes were compared for similarities and differences and sorted into sub-categories and categories. The analysis went back and forth between the interviews, codes, sub-categories and categories to validate the results. Each step of the analysis was discussed by the researchers at regular meetings until consensus was achieved.

### Ethical approval

The study was approved by the Regional Ethical Review Board in Uppsala (Dno. 2012/541) and conforms to the principles outlined in the Declaration of Helsinki [48]. The RNs received written and verbal information on the study and signed a written informed consent prior to each interview.

### Results

All FGIs had a high level of interaction between the RNs in the process of comparing and contrasting views, and constructing meaning about the topic for the interviews. Intra-group agreement was high in all interviews. Inter-group disagreement was apparent between FGIs in the hospital and FGIs in the PHCCs. The nature of this inter-group disagreement was that the RNs in PHCCs had contact with family caregivers to patients with HF over the telephone, at patients' routine checks for blood pressure and during their treatment for leg ulcers. In these contacts, patients' HF condition was not consciously reflected upon until asked about in the study's FGIs. RNs' in the PHCCs perceptions derived from their present work but also from their previous work in home health care, the latter having been the responsibility of PHCCs until two years previously.

Four categories and nine sub-categories emerged in the analysis process (Table 3). The results are presented according to the two content areas identified by the a priori study aims: "Registered nurses' perceptions about family caregivers' situation" and "Registered nurses' interventions to improve family caregivers’ situation", along with their underlying categories and sub-categories with interview extracts. Both content areas comprise RNs' perceptions about patients and family caregivers on an interpersonal level, and RNs' perceptions about study setting specific care issues on an organizational level.

**Table 3:** Overview of categories and sub-categories in content areas 1 and 2.

**Content area 1. Registered nurses' perceptions about family caregivers' situation**	
**Categories**	**Sub-categories**
To be unburdened	1.Relieved of medical responsibility
	2.Respite from caregiving
	3.Continuity in health care contacts
To comprehend the heart failure condition and its consequences	1.Knowledge and understanding
	2.Relationship within the family
**Content area 2. Registered nurses' interventions to improve family caregivers' situation**	
**Categories**	**Sub-categories**
Individualized support and information	1.Supportive counselling
	2.Information
Bridging contact	1.Cooperation and collaboration
	2.Extended arenas for support and information

### Registered nurses' perceptions about family caregivers' situation

#### To be unburdened: 1. Relieved of medical responsibility

RNs perceived family caregivers as prime carriers of the medical responsibility for their relative. Some family caregivers were hesitant to leave their relative at home alone in case the HF condition worsened.

FGI 6:

*To be the one who takes care of a patient with heart failure*, *well that is a great responsibility* (P 1). *Very much so* (P 2). *And often they feel insecure in that situation* (P 1). *Very burdensome*, *I can imagine*, *burdensome and … they feel they have to be at home*, *to always check up (on their relative)* (P 2).

Family caregivers were considered to handle the responsibility with varying degrees of worry and uncertainty. The variations were primarily seen as related to the severity of their relative’s HF, the relative's lack of adherence and its consequences and the complexity of the medication regimen.

FGI 5:

*Clearly they are worried*, *but it depends on what state the patient is in* (P 2).

Worry and uncertainty were also seen as related to making the right medical decisions such as deciding on when to contact health care and how to handle worsening of HF symptoms. RNs agreed upon that when family caregivers did not want to carry the medical responsibility then they should not have to.

FGI 2:

*… they often need to talk about what happened at home prior to the hospitalization* (P 3). *They (family caregivers) need to confirm that they did the right thing*, *both at home and when coming here* (P 1).

To make a wrong decision left them with a bad conscience and in a state of remorse. As most family caregivers were perceived to feel a duty to care, bad conscience was also a consequence if they failed in this duty.

FGI 1:

*The caregivers feel that they must do right*, *which creates unrest and guilt if they do wrong*. *But they don't dare to say that they are not able to care because they feel obligated to help their relative otherwise they get a bad conscience* (P 4).

#### To be unburdened: 2. Respite from caregiving

RNs in the hospitals perceived the patient's hospitalization to be a temporary respite from caregiving for the family caregivers. The hospitalization was at times an eye-opener on their untenable home situation. The hospital admittance offered them a chance to focus on their own well-being, and a common request was to let their relative stay for an extra day or two.

FGI 1:

*When they are here and have left the patient they can say “I have taken care of him for weeks*, *now I have to go home and rest*, *get some sleep*. *Is it OK that I leave now*?*”* (P 1).

RNs in the PHCCs perceived that family caregivers toned down their description of their strenuous situation.

FGI 4:

*I believe that this group of family caregivers (to patients with HF) doesn’t talk about how poorly they feel; they probably need much more support* (P 1). *Yes*, *the focus is on the patient and they (family caregivers) are forgotten about* (P 2).

An inter-group agreement between the FGIs in the PHCCs concerned their acknowledgment of family caregivers' burdensome situation. The RNs wanted to improve their situation, whether it concerned practical issues or lack of knowledge or emotional discomfort, but felt hindered in doing so, primarily due to time-constraints.

#### To be unburdened: 3. Continuity in health care contacts

There was an overall inter- and intragroup agreement among RNs that family caregivers preferred the continuity of a permanent health care contact, whether they needed reassurance and relief from their worry or information on practical issues, or the HF condition. Easily accessible contact channels meant security for the family caregivers.

The hospitals' nurse-led HF clinics, or medical outpatient clinics with designated time for follow-up of patients with HF, had routines for return visits besides continuous follow-ups by telephone. These routines were considered to prevent unnecessary emergency visits due to worry and uncertainty on whom to turn to for help. RNs in the hospitals conveyed frustration over deficiencies in the care continuum regarding patients who were not registered to receive home health care from the municipality. RNs perceived a disagreement between county council and municipality about the financial responsibility for home care and home health care services. This disagreement was considered by the RNs to be an inherent obstacle in the health care organisation that complicated the situation for family caregivers.

FGI 2:

*Yes*, *if a patient needs help with the support stockings*, *or a check up on the daily weight*, *well home care doesn’t do that since it’s medical and home health care (in the municipality) is now so difficult to get*, *so we have to encourage the family caregivers to do the job* (P 3).

The above-mentioned compromised continuity with the municipality's home health care was also discussed in the PHCCs, where it was reported to function satisfactorily.

FGI 5:

*We have a close collaboration with the RNs in the municipality’s home health care*. *These patients go back and forth; when they don’t manage to come here they receive home health care and later they might get better again and come back*. *So we need to collaborate*, *otherwise it doesn’t work* (P 2).

In the PHCCs, the RNs primarily came in contact with family caregivers by telephone. RNs acknowledged the advantages of having easily accessible and clear contact channels with patients with HF and their family caregivers but no such routines existed. With high demands on telephone accessibility, the RNs were required to quickly focus on the patient's problem rather than the family caregiver's.

#### To comprehend the heart failure condition and its consequences: 1. Knowledge and understanding

RNs in the hospitals perceived that family caregivers lacked knowledge and understanding of their relative's quick deterioration in HF, thus prompting emergency care. They also lacked knowledge and understanding of the need for the large quantities of HF medications and intravenous diuretic treatment. Upon readmission, family caregivers at times expressed irritation with the health care's inability to adjust medications so as to avoid unwanted readmissions. There was an overall agreement among RNs in the hospitals that family caregivers wanted to learn how to early detect signs of deterioration in their relative to avoid readmissions. The most appropriate occasion to equip them with adequate knowledge was at hospital discharge. An inter-group agreement of all FGIs was apparent on the need for RNs to share their knowledge of HF to improve family caregivers' ability in early detection and reduce worry and uncertainty over the worsening of HF. Family caregivers' feelings of uncertainty and worry were considered as main hindrances to their intake of new knowledge.

FGI 1:

*… they need security I think*, *the security of knowing … recognizing the symptoms* (P 3). *Yes*, *one can notice that many questions are repeated*, *and their thoughts get blocked from hearing one thing and then they don't hear the rest* (P 2).

Family caregivers were perceived as not wanting to understand that HF was a progressive condition and a steadily deteriorating process. Instead, they viewed HF as a condition that could be kept in complete control with the appropriate medication. On the other hand, the RNs were unwilling to inform the family caregivers and patients that HF was a serious and deteriorating condition as they did not want to deprive family caregivers and patients of their hope and positive spirit.

Family caregivers and their relatives rarely came together to visits with the physician or RN, whether in hospital or the PHCC. However, on the rare occasions when they did, it was obvious to the RNs that important information from the patient's last visit had not been shared with their family caregiver.

FGI 3:

*… at times when I've met the patient*, *and they have a spouse with them*, *I ask them both about weighing*, *and the spouse doesn’t understand why*, *even though I have told the patient how important it is*. *No*, *I don’t think the patient tells* (P 1).

#### To comprehend the heart failure condition and its consequences: 2. Relationship within the family

There was an inter-group agreement among the RNs in the hospitals on the presence of incongruence in the relationship. Incongruence was considered to stem from lack of knowledge and understanding of the severity of HF and on how to manage it and was particularly noticeable in relation to seeking emergency care. While the family caregivers urged their relatives to seek care, the relatives downplayed their symptoms of deterioration, thus delaying treatment. RNs considered the family caregivers' assessment of symptoms to be the most correct. RNs also observed family caregivers' irritation with their relative’s lack of energy or even laziness. This irritation, which was a result of family caregivers' lack of knowledge and understanding of the HF condition, had a negative impact on the relationship.

FGI 3:

That the spouse might understand that he (the patient) actually can’t put on his own shoes. Maybe he did it two days ago, but now he absolutely cannot or maybe they (family caregivers) say "so why do you sit here and sleep all day".

RNs found it sad to see how the family caregivers isolated themselves and adjusted their own energy level and mood to tune in with their relative's.

FGI 1:

*It’s incredibly sad for the person who is healthy*, *I often hear “Well*, *I’ll just stay at home" (P 2)*. *Mm*, *but after a long life of marriage*, *you become as one*, *if the sick one doesn’t want to go out and meet friends*, *then you choose to stay at home* (P 3).

RNs identified "team spirit", or loyalty, within the relation as a key factor in achieving good treatment results and congruence.

FGI 2:

"*As a family caregiver I work* *together* *with the one who is ill*. *I won't drink big glasses of water in front of the person who himself cannot*. *Instead I'll just take an extra glass myself when I'm doing the dishes*.*" They work* *with* *(in self-care) instead of* *against*. *I think these couples have the best chances to succeed* (P 3).

RNs in a PHCC observed how patients took the family caregivers' care for granted and did not realize the long-term demands they placed on their family caregiver. Neither was the family caregivers’ need for external support understood nor easily approved of by the patient. RNs described how family caregivers were rarely explicit about their situation and needs when their relative was present, but only hinted at these matters so as not to hurt their feelings or divert focus from their relative. When RNs spoke alone with the family caregivers they explicitly expressed their fatigue and need for practical support to the RNs.

FGI 6:

*The family caregiver says that they maybe need a little help*, *then you notice that the patient gets worried about having people around at home* (P 5). *"No*, *it's fine*, *it’s fine as it is” says the patient without really seeing that the family caregiver is trying to convey …* (P 1). *“… that I need some help”* (P 2). *With body language they say it as well*, *because they don’t always dare to speak it out loud*. *One sees the worry in the patient's eyes over being cared for by others but*, *the patient does not think about how demanding this is for their caregiver* (P 5).

### Registered nurses' interventions to improve family caregivers’ situation

#### Individualized support and information: 1. Supportive counselling

RNs considered themselves as responsible for initiating the discussion with family caregivers on practical and emotional issues. Family caregivers were rarely inclined to do so themselves why family caregivers: *…* *needed* *help to* *ask* *for help* (FGI 3, P 2). None of the RNs in hospitals and PHCCs routinely invited family caregivers to private supportive counselling although RNs overall agreed on the need to do so. They saw an opportunity for family caregivers to be more open about their needs when not being in the presence of their relative. RNs in the hospitals suggested to call the family caregivers while their relative was in hospital, or to encourage the family caregivers to come to the RNs' office while they were visiting in the hospital.

RNs in the PHCCs suggested to speak with the family caregivers when their relative was resting in an adjacent room prior to measure their blood pressure. RNs were explicit on their responsibility to support family caregivers in their quest for help but always with respect for the patient's wishes.

FGI 6:

*It’s important not to override the person who is ill* (P 2). *No*, *of course not* (P 5). *One needs to tread carefully … if the patient says “everything is fine” but you see that the family caregiver needs help*, *you have to find the appropriate level of counselling* (P 2).

Family caregivers were considered to greatly benefit from clear and accessible contact channels. There was an overall inter-group agreement on the importance of offering the continuity and security of a RN as a permanent health care contact to call whenever needed. In the hospitals, the RN in the nurse-led HF clinic was such a contact.

FGI 3:

*I notice that the family caregivers are more content*, *feel more secure* (P 2).

Although the PHCCs lacked RNs educated in cardiac care and routines for follow-up of heart failure patients, a similar system of continuity was agreed upon as possible. The following extract illustrates a feasible "how to".

FGI 4:

*There are many who are at home in uncertainty* (P 4). *There are so many of us nurses*, *and they would feel more secure if they had a name of someone*, *"Can I talk to Kerstin*, *is she there*? *She knows us"* (P 1). *That they knew to whom they could turn* (P 3).

#### Individualized support and information: 2. Information

RNs in the hospitals described themselves as advocates of self-care. It was their responsibility to help and guide the patient and their family caregiver to find new ways to live as normally as possible. RNs in the nurse-led HF clinic in one hospital were routinely notified as patients with HF were admitted to the medical ward.

For informational purposes, RNs encouraged joint health visits. When patients preferred to come to health visits on their own, RNs tried to convince them of the advantages of bringing their family caregiver.

FGI 2:

*The patients leave their family caregiver in the waiting room*, *the patients want to come in on their own*. *But then I say “bring your family caregiver with you*, *it’s much easier to remember when you’re two and they will understand you better*, *like when you’re tired some days”*, *then they change their mind and invite the family caregiver to follow them in* (P 1).

Family caregivers’ presence created a win-win situation as they had expert knowledge about their relative's health status in addition to gaining important knowledge on HF and self-care. They also helped the RNs to understand reasons for deterioration, as well as compensating for their relative's eventual cognitive impairment. A frequent statement in several of the FGIs was that: … *two pairs of ears hear more than one*. Adherence to self-care was believed to be higher if both parties heard and understood the reasoning behind the self-care regimen. Also, the patient sought health care earlier when having a well-informed and motivated family caregiver by their side.

FGI 2:

*Often the symptoms come insidiously*, *very slowly*. *And the one with HF may not be aware of the deterioration*, *but the family caregiver sees that the number of pillows is increasing and the daily walk becomes more and more strenuous* (P 3).

Information and knowledge on self-care should be conveyed in a comprehensive manner. It had to be basic, honest and explanatory to increase motivation. It also had to be repeated several times and complemented with booklets. If the information was too simplified, the patient and their family caregiver would not understand the severity of HF and the need for self-care. On the other hand, if delivered with exaggerated directness and too much at the same time, it caused worry and lack of motivation for self-care. To inform on the severity and progression of the HF condition in a straightforward yet cautious manner was a difficult balancing act.

FGI 1:

*To say “this is serious” and explain in a simple way how to avoid deterioration* (P 2). *It has to do with compliance; one cannot trivialize the condition or maybe they don’t follow treatment guidelines*, *they don’t take it seriously* (P 1). *They need to understand why they take the medication* (P 4). *A simple explanation suffices*, *and it must be explained to family caregivers too* (P 2). *Yes*, *because then they can remind and help at home* (P 4).

Not least important, information from the different RNs and physicians had to be consistent to avoid that patients and family caregivers became confused from mixed messages.

#### Bridging contact: 1. Cooperation and collaboration

Cooperation between the nurse-led HF clinic, or medical outpatient clinic, and various other units in the hospitals was considered to be accessible and satisfactory by RNs in the hospitals.

The advanced home health care unit in one hospital strived to work closely with RNs in the medical outpatient clinic concerning patients whose HF condition fluctuated in severity. The alternative for these patients and their family caregivers was otherwise to seek repeated emergency care. Another way to bypass the strenuous emergency unit care was to have an established direct line to the cardiac ward, thus avoiding long waiting hours and suffering for the patients and family caregivers. Also, the medical outpatient clinic provided diuretic intravenous treatment when symptoms of deterioration were identified early, thus again bypassing the emergency unit.

FGI 3:

*The severity of HF varies over time*, *so at times they come here (medical outpatient clinic)*, *get diuretics intravenously and then return back home*. *We work to prevent these re-hospitalizations*, *this yo-yo back and forth* (P 3).

There was an inter-group agreement among the RNs in the hospitals on the need for improved collaboration with the PHCCs in the county council, and home care and home health care in the municipality. Collaboration functioned fairly well providing the patients were stable in their HF status and capable of coming to the PHCC for follow-up, or if they were registered in the municipality’s home health care. Alas, collaboration was more difficult when a patient’s health status was poor, but not poor enough to qualify for home health care. Temporary home health care visits on behalf of another caregiver than primary care, for example the hospital, was not included in the municipalisation of home health care. Thus, when the hospital recommended follow-up of these patients in their homes, but the physician in the PHCC in primary care did not, a financial and responsibility dilemma between county council and municipality arose. To solve this dilemma, RNs in the hospitals suggested three interventions: to expand on the hospital-based advanced home health care services; develop a closer collaboration with the family caregivers; and establish a coordinated individual care plan as a basis for collaboration to prevent re-hospitalizations.

FGI 1:

*She has come here several times*, *why*? *If there are several different caregivers*, *well then we establish a care plan “if she gains weight by two kilos*, *we do this”*, *“if this or that happens*, *we call here” and so on*. *Simple directions but most importantly*, *who is responsible for the patient if we (RNs in the nurse-led HF clinic) are no longer involved* (P 2). *And there is a VIP telephone line into the primary health care centre; they don’t have to wait in a queue* (P 4).

The need for a coordinated individual care plan at hospital discharge as a preparation before returning home was also discussed by the RNs in the PHCCs.

FGI 6:

*It's about information and collaboration*, *they (all involved at hospital discharge) have to draw up an individual plan so that everyone is aware that "this is how it's gonna be"* (P 1).

RNs also emphasized the importance of close collaboration with the RNs in the municipality since patients went back and forth between care in the PHCC and receiving care at home in the municipality's home health care.

#### Bridging contact: 2. Extended arenas for support and information

RNs in the hospitals suggested several arenas for family caregivers to receive support and information from each other and from health care professionals. Hospital-based open lectures with refreshments on a regular basis was one, another was support groups in the hospital or in the community, preferably in combination with education on HF. One nurse-led HF clinic had regularly booked exercise and swimming groups for patients with HF, to which RNs suggested family caregivers should be invited. RNs perceived the HF condition to be relatively “unknown” among people in general. Thus, RNs suggested a broad-based information day on HF in the community to bring attention to the condition. RNs in the hospitals also considered the knowledge of HF care to be inadequate among the municipality's RNs working in home health care, home care personnel, and the RNs in the PHCCs. RNs in the hospitals suggested education for all three groups which could lead to increased support for the family caregivers.

In the PHCCs, RNs acknowledged their own lack of knowledge of HF care. There was an inter-group disagreement on reasons for this deficiency. Either because PHCCs did not have designated time to care for patients with HF or had a general lack of time, or as a consequence of "losing" their home health care patients with heart failure to the municipality two years ago. If patients with HF were identified earlier in primary care it was perceived as indirectly supportive for the family caregivers. Two arenas for early identification were suggested: when taking the patient's blood pressure, and during the routine health screening of persons ≥ 75 years old.

FGI 6:

*We have something called GRP (Geriatric Risk Profile*, *an assessment instrument used in health screening) and it's probably a large part of those who come who have HF*. *We go through their medication and sometimes they have their family caregivers with them*. *So*, *there we can identify them; starting from what we can do now* (P 3).

## Discussion

RNs in both hospitals and PHCCs perceive family caregivers' situation as burdensome, above all worry and uncertainty permeate several categories in the result. RNs in the PHCCs avoid to ask about family caregivers' situation as they lack time and resources to perform appropriate nursing interventions besides referring the family caregivers to other professionals. Still, RNs acknowledge the need for a supportive intervention and suggest to routinely provide a named RN to whom the family caregiver and their relative can telephone whenever needed in order to mitigate worry and uncertainty. This continuity and security of a permanent health care contact by telephone is considered a feasible and sustainable intervention in the PHCCs. RNs are aware of that contact via telephone was not optimal, and also shown to be inferior to in-person communication with HF patients [26], but to do one's best in the given circumstances has to suffice when the alternative is no continuity at all. The continuity of one named person, is also in line with the Patient Act [49] stating that a permanent health care contact should be appointed for the patient if requested, or if necessary to meet the needs of security, continuity, coordination and safety.

Family caregivers' worry and uncertainty is well documented in the chronic illness and HF literature and is associated with family caregivers' lack of knowledge, the severity and unpredictable course of HF, and family caregivers' need of involvement in the planning and implementation of their relative's health care [15,25,50]. In this study, the RNs perceive the two former reasons but not the latter concerning the importance of family caregiver involvement, which is problematic as there exists an important link between RNs' perceptions about family caregivers' situation and RNs' provision of nursing care [35]. Constraining and facilitating beliefs have a predictive effect on perceptions and actions, and they impact upon both the RNs and the family involved [51]. RNs do encourage joint health visits in the hospital, especially at hospital discharge, but not for the benefit of the family caregiver or the family as a unit but rather to improve on patient's self-care. Currently, most HF nursing interventions primarily focus on patients to improve outpatient self-care [37], although research emphasizes that nursing interventions should recognize the importance of family care relationships to achieve optimal outcomes [52]. It is also argued that Family-centred care should be an integral part of RNs’ work, especially when caring for patients with severe and long-term medical conditions, and their family caregivers [53,54]. As RNs in the present study seemingly do not possess the tools to provide family caregivers with the opportunity of involvement in the planning and implementation of their relative's health care, family conversation may prove to be such a tool. RNs in the hospitals who have designated time for follow-up of patients with HF can play an important role as partners in family conversations that explore the patient's and their family caregiver's experiences and beliefs and promote health in a trusting relationship [55,56,57]. Family conversation in HF nursing care has shown to reduce RNs' feelings of powerlessness [56] and facilitate more constructive relationships with the families [57,58]. Family conversation may be particularly helpful to RNs when counselling on the serious prognosis and deteriorating process of HF, on family caregivers' bad conscience, their feelings of having a duty to care and their social isolation [53,59]. Family-centred care requires that all parties are equal and learn about each other's beliefs and competencies. No one’s experiences take precedence over the others' experiences and competencies as they are all given equal importance [53,59].

RNs in hospitals consider the patient's and their family caregiver's lack of knowledge and understanding to cause incongruence in their relationship. This incongruence is particularly pronounced when seeking emergency care. The HF literature shows that incongruent relationships report more conflict, psychosocial stress and tension that arise in relation to self-care and deciding on when to seek medical treatment [60,61]. In the PHCCs, RNs perceive incongruence in relation to how the patients neither understands the demands they put on the family caregivers, nor family caregivers' need of external support. Overall, RNs describe how difficult it is for family caregivers to voice their burdensome situation and request help in the presence of their relatives. Therefore RNs suggest to occasionally counsel and support family caregivers in private despite also acknowledging that private consultations potentially compromise the integrity of the patient. In the explorative study by Lum et al. [37] relationship quality was positively associated with HF caregiver benefit and negatively associated with HF caregiver burden. Moreover, a recent integrative review [62] concludes that asking about relationship quality may yield important information about the health and well-being of both the HF patients and their family caregivers. It thus seems essential to be aware of and address congruence and relationship quality within the family. One of the strategies suggested in the literature is the construct of "Shared care" [36]. This recognizes the unique importance of communication about the shared illness experience, the decision-making on how to respond to the illness, and reciprocity skills in care activities. Again, the RNs in the hospitals who have designated time for patients with HF can use "Shared Care" to assess communication and decision-making in the relation between patient and family caregiver. The use of "Shared Care" has been shown to improve self-care in patients with HF, and has also led to improvements in relationship quality and health for family caregivers [52].

Several RNs in both hospitals and PHCCs voiced their frustration when addressing the deficient collaboration between the two health authorities on the care for patients with HF. RNs considered it necessary to have a coordinated individual care plan, which includes the needed patient interventions and the authority responsible for the respective intervention. RNs perceptions are supported by the Social Services Act [63] and the Health Care Act [64] stating that a coordinated individual care plan shall be jointly established by the county council and municipality when a patient is in need of care and support from both health authorities.

### Strengths and limitations

All FGIs were homogenous and the participants were comfortable in expressing their thoughts and perceptions. However, some of the participants were more verbal than others and there is a slight surplus of extracts from them as they were able to summarize what several other participants said, but in a more straightforward way. Only a minority of the participating RNs had previously reflected on the situation of family caregivers. Therefore, the focus group format turned out to be particularly suitable in this study as the group dynamic assisted the participants to actively clarify their views, which would have been less likely to occur in individual interviews. The homogeneity may also have been a limitation as RNs in one health unit missed the opportunity to share, discuss and understand the perceptions and feasible interventions of RNs from another health unit in the health organisation. On the other hand, RNs in PHCCs may have been inhibited in the FGIs by the hospital RNs' relatively larger expertise and daily experience in HF nursing.

The RNs did not have first-hand experience of the topic for the interviews; instead, they contributed with their perceptions about the situation of others. This “meta-level” perspective calls for caution when analysing the data material, which is why the researchers chose to remain on a descriptive, manifest level of the text.

Another concern was the discontentment of the RNs in two of the three PHCCs due to the recent shift of responsibility for home health care from county council to the municipality. Thus, they concluded that they had "lost" contact with patients with HF and their family caregivers. However, these groups were particularly rich in their associations with their colleagues’ perceptions and experiences. They reflected upon the arenas of contact they still had left and were creative in suggesting extended arenas for support and information in their future work. Moreover, the scope of perceptions and interventions to improve family caregivers' situation would probably have been larger if RNs in the municipal home health care were interviewed. Furthermore, the transferability of this study's results should be interpreted with the two different levels of RNs' perceptions in mind. On the organizational level, RNs' perceptions about the financial and responsibility dilemma between county council and municipality are highly study setting specific and thus reduce the transferability of our results. The perceptions of RNs about patients and family caregivers on an interpersonal level are more likely to be transferable to other patient and family caregiver groups and a variety of health care organizations.

## Conclusions

RNs perceive family caregivers' situation as burdensome and characterized by worry and uncertainty. In the PHCCs, the continuity and security of an RN as a permanent health care contact was considered an important and sustainable intervention to better care for family caregivers' worry and uncertainty. In the nurse-led heart failure clinics in hospitals, RNs can provide family caregivers with the opportunity of involvement in the planning and implementation of their relative's health care and address issues of congruence and relationship quality within the family through the use of "Shared care" and or Family-centred care. RNs consider it necessary to have a coordinated individual care plan as a basis for collaboration between the county council and the municipality.

## References

[1] Yancy CW, Jessup M, Bozkurt B, Butler J, Casey DE, Drazner MH, et al ACCF/AHA guideline for the management of heart failure: a report of the American College of Cardiology Foundation/American Heart Association Task. JACC. 2013;62(16): e147–239. 23747642 10.1016/j.jacc.2013.05.019

[2] McMurray JJ, Adamopoulos S, Anker SD, Auricchio A, Böhm M, Dickstein K, et al ESC Guidelines for the diagnosis and treatment of acute and chronic heart failure 2012: the Task Force for the Diagnosis and Treatment of Acute and Chronic Heart Failure 2012 of the European Society of Cardiology. Eur J Heart Fail. 2012;14(8): 803–869. 22828712 10.1093/eurjhf/hfs105

[3] Roger VL. The heart failure epidemic. Int J Environ Res Public Health. 2010;7(4): 1807–1830. 10.3390/ijerph7041807 20617060 PMC2872337

[4] SoS, Socialstyrelsen [In Swedish: NBHW, National Board of Health and Welfare]. Hjärtsvikt: Vetenskapligt underlag för Nationella riktlinjer för hjärtsjukvård 2008 [In Swedish: Heart failure: Scientific basis for national guidelines for cardiac care 2008]. Stockholm: Socialstyrelsen; 2008.

[5] McMurray JJ, Stewart S. Epidemiology, aetiology and prognosis of heart failure. Heart. 2000;83(5): 596–602. 10768918 10.1136/heart.83.5.596 PMC1760825

[6] Annema C, Luttik ML, Jaarsma T. Reasons for readmissions in heart failure: Perspectives of patients, caregivers, cardiologists, and heart failure nurse. Heart Lung. 2009;38(5): 427–434. 10.1016/j.hrtlng.2008.12.002 19755193

[7] Pressler SJ, Gradus-Pizlo I, Chubinski SD, Smith G, Wheeler S, Sloan R, et al Family caregivers of patients with heart failure. A longitudinal study. J Cardiovasc Nurs. 2013;28(5): 417–428. 10.1097/JCN.0b013e3182563877 22760173

[8] Strömberg A. The situation of caregivers in heart failure and their role in improving patient outcomes. Curr Heart Fail Rep. 2013;10(3): 270–275. 10.1007/s11897-013-0146-8 23857163

[9] Bidwell JT, Vellone E, Lyons KS, D'Agostino F, Riegel B, Juárez-Vela R, et al. Determinants of heart failure self-care maintenance and management in patients and caregivers: A dyadic analysis. Res Nurs Health. 2015 Aug 20. [Epub ahead of print] 10.1002/nur.21675 PMC4654948 26355702

[10] Gallagher R, Luttik ML, Jaarsma T. Social support and self-care in heart failure. J Cardiovasc Nurs. 2011;26(6): 439–445. 10.1097/JCN.0b013e31820984e1 21372734

[11] Salyer J, Schubert CM, Chiaranai C. Supportive relationships, self-care confidence, and heart failure self-care. J Cardiovasc Nurs. 2012;27(5): 384–393. 10.1097/JCN.0b013e31823228cd 22048619

[12] Buck HG, Harkness K, Wion R, Carroll SL, Cosman T, Kaasalainen S, et al Caregivers’ contributions to heart failure self-care: a systematic review. Eur J Cardiovasc Nurs. 2015;14(1): 79–89. 10.1177/1474515113518434 24399843

[13] Clark AM, Reid ME, Morrison CE, Capewell S, Murdoch DL, McMurray JJ. The complex nature of family care in home-based heart failure management. J Adv Nurs. 2008;61(4): 373–383. 10.1111/j.1365-2648.2007.04527.x 18234035

[14] Clark AM, Spaling M, Harkness K, Spiers J, Strachan PH, Thompson DR, et al Determinants of effective heart failure self-care: a systematic review of patients' and caregivers' perceptions. Heart. 2014;100(9): 716–721. 10.1136/heartjnl-2013-304852 24548920

[15] Kang X, Li Z, Nolan MT. Family caregivers' experiences of caring for patients with chronic heart failure. J Cardiovasc Nurs. 2011;26(5): 386–394. 10.1097/JCN.0b013e3182076a69 21263337 PMC8976444

[16] Kosiborod M, Soto GE, Jones PG, Krumholz HM, Weintraub WS, Deedwania P, et al Identifying heart failure patients at high risk for near-term cardiovascular events with serial health status assessments. Circulation. 2007;115(15): 1975–1981. 17420346 10.1161/CIRCULATIONAHA.106.670901

[17] Whittingham K, Barnes S, Gardiner C. Tools to measure quality of life and carer burden in family carers of heart failure patients: a narrative review. Palliat Med. 2013; 27(7): 596–607. 10.1177/0269216313477179 23442879

[18] Luttik ML, Jaarsma T, Veeger NJ, van Veldhuisen DJ. For better and for worse: quality of life impaired in HF patients as well as in their partners. Eur J Cardiovasc Nurs. 2005;4(1): 11–14. 15718187 10.1016/j.ejcnurse.2004.12.002

[19] Årestedt K, Saveman BI, Johansson P, Blomqvist K. Social support and its association with health-related quality of life among older patients with chronic heart failure. Eur J Cardiovasc Nurs. 2013;12(1): 69–77. 10.1177/1474515111432997 22457369

[20] Trivedi RB, Piette J, Fihn SD, Edelman D. Examining the interrelatedness of patient and spousal stress in heart failure: conceptual model and pilot data. J Cardiovasc Nurs. 2012;27(1): 24–32. 10.1097/JCN.0b013e3182129ce7 21743348

[21] Ågren S, Evangelista L, Strömberg A. Do partners of patients with chronic heart failure experience caregiver burden? Eur J Cardiovasc Nurs. 2010;9(4): 254–262. 10.1016/j.ejcnurse.2010.03.001 20598946 PMC3180929

[22] Pihl E, Jacobsson A, Fridlund B, Strömberg A, Mårtensson J. Depression and health-related quality of life in elderly patients suffering from heart failure and their spouses: a comparative study. Eur J Heart Fail. 2005;7(4): 583–589. 15921798 10.1016/j.ejheart.2004.07.016

[23] Ågren S, Evangelista L, Hjelm C, Strömberg A. Dyads affected by chronic heart failure: a randomized study evaluating effects of education and psychosocial support to patients and their partners. J Cardiac Fail. 2012;18(5): 359–366. 10.1016/j.cardfail.2012.01.014 PMC3381875 22555264

[24] Pihl E, Fridlund B, Mårtensson J. Spouses' experiences of impact on daily life regarding physical limitations in the loved one with heart failure: a phenomenographic analysis. Can J Cardiovasc Nurs. 2010;20(3): 9–17. 20718235

[25] Gusdal AK, Josefsson K, Thors Adolfsson E, Martin L. Informal Caregivers' Experiences and Needs when Caring for a Relative with Heart Failure: An Interview Study. J Cardiovasc Nurs. 2014 11 21. [Epub ahead of print] 10.1097/JCN.0000000000000210 25419945

[26] Sochalski J, Jaarsma T, Krumholz HM, Laramee A, McMurray JJ, Naylor MD, et al What works in chronic care management: the case of heart failure. Health Aff. 2009; 28(1): 179–189. 10.1377/hlthaff.28.1.179 19124869

[27] McAlister F, Stewart S, Ferrua S, McMurray J. Multidisciplinary strategies for the management of heart failure patients at high risk for admission - a systematic review of randomized trials. J Am Coll Cardiol. 2004;44(4): 810–819. 15312864 10.1016/j.jacc.2004.05.055

[28] Roccaforte R, Demers C, Baldassarre F, Teo KK, Yusuf S. Effectiveness of comprehensive disease management programmes in improving clinical outcomes in heart failure patients. A meta-analysis. Eur J Heart Fail. 2005;7(7): 1133–1144. 16198629 10.1016/j.ejheart.2005.08.005

[29] Strömberg A, Mårtensson J, Fridlund B, Levin LÅ, Karlsson JE, Dahlström U. Nurse-led heart failure clinics improve survival and self-care behaviour in patients with heart failure: results from a prospective, randomized trial. Eur Heart J. 2003;24(11): 1014–1023. 12788301 10.1016/s0195-668x(03)00112-x

[30] Takeda A, Taylor SJ, Taylor RS, Khan F, Krum H, Underwood M. Clinical service organisation for heart failure. Cochrane Database Syst Rev, 9, D002752; 2012. 10.1002/14651858.CD002752.pub3 22972058

[31] Mårtensson J, Dahlström U, Johansson G, Lernfelt B, Persson H, Willenheimer R, for the Heart Failure Working Group of the Swedish Society of Cardiology. Nurse-led heart failure follow-up in primary care in Sweden. Eur J Cardiovasc Nurs. 2009;8(2): 119–124. 19046656 10.1016/j.ejcnurse.2008.10.002

[32] Strömberg A. Diagnostik och behandling av kronisk hjärtsvikt – Bakgrunds-dokumentation: Strukturerad vård ger bättre effektivitet [In Swedish: Diagnostics and treatment of chronic heart failure - Background documentation: Structured care is more efficient]. Information från Läkemedelsverket, 1:2006 [In Swedish: Information from the Medical Products Agency, 1:2006]. Stockholm: Läkemedelsverket; 2006.

[33] McDonagh TA, Blue L, Clark AL, Dahlström U, Ekman I, Lainscak M, et al European Society of Cardiology Heart Failure Association Committee on Patient care. European Society of Cardiology Heart Failure Association Standards for delivering heart failure care. Eur J Heart Fail. 2011;13(3): 235–241. 21159794 10.1093/eurjhf/hfq221

[34] Jaarsma T, Strömberg A. Heart Failure Clinics Are Still Useful (More Than Ever?). Can J Cardiol. 2014;30(3): 272–275. 10.1016/j.cjca.2013.09.022 24468418

[35] Wright LM, Leahey M. Nurses and families: a guide to family assessment and intervention 6th ed. Philadelphia, PA: F.A. Davis Company; 2013.

[36] Sebern M. Shared care, elder and family member skills used to manage burden. J Adv Nurs. 2005;52(2): 170–179. 16164478 10.1111/j.1365-2648.2005.03580.x

[37] Lum HD, Lo D, Hooker S, Bekelman DB. Caregiving in heart failure: Relationship quality is associated with caregiver benefit finding and caregiver burden. Heart Lung. 2014; 43(4): 306–310. 10.1016/j.hrtlng.2014.05.002 24992881 PMC5711727

[38] Holloway I, Wheeler S. Qualitative research in nursing and healthcare 3rd ed. Oxford: Wiley-Blackwell; 2010.

[39] Krueger RA, Casey MA. Focus groups 5th ed. Thousand Oaks, CA: Sage Publications; 2015.

[40] McLafferty I. Focus group interviews as a data collecting strategy. J Adv Nurs. 2004;48(2): 187–194. 15369499 10.1111/j.1365-2648.2004.03186.x

[41] SKL, Sveriges kommuner och landsting [In Swedish: SALAR, Swedish Association of Local Authorities and Regions]. (2015). Available: http://skl.se/tjanster/englishpages/municipalitiescountycouncilsandregions.1088.html Accessed 15 March 2015.

[42] SwedeHF, The Swedish Heart Failure Registry. Uppsala: Rikssvikt. Retrieved April, 2, 2015, from http://www.ucr.uu.se/rikssvikt/

[43] Kvale S, Brinkmann S. Den kvalitativa forskningsintervjun [In Swedish: The qualitative research interview]. Lund: Studentlitteratur; 2009.

[44] Molloy GJ, Johnston DW, Witham MD. Family caregiving and congestive heart failure. Review and analysis. Eur J Heart Fail. 2005;7(4): 592–603. 15921800 10.1016/j.ejheart.2004.07.008

[45] Graneheim UH, Lundman B. Qualitative content analysis in nursing research: Concepts, procedures and measures to achieve trustworthiness. Nurse Educ Today. 2004;24(2): 105–112. 14769454 10.1016/j.nedt.2003.10.001

[46] Krippendorff K. Content Analysis: An Introduction to its Methodology 3rd ed. Thousand Oaks, CA: Sage Publications; 2013.

[47] NVivo10. NVivo software program London: QSR International (UK) Limited; 2012.

[48] WMA, World Medical Association. Declaration of Helsinki - Ethical principles for medical research involving human subjects. 2015. Available: http://www.wma.net/en/30publications/10policies/b3/. 10.1191/0969733002ne486xx 16010903

[49] SFS, Svensk författningssamling 2014:821 [In Swedish: Swedish Code of Statutes]. Patientlag [In Swedish: Patient Act]. Stockholm: Socialdepartementet [In Swedish: Ministry of Social Affairs]. Available: http://www.riksdagen.se/sv/Dokument-Lagar/Lagar/Svenskforfattningssamling/Patientlag-2014821_sfs-2014-821/#K7P1.

[50] Hartmann M, Bäzner E, Wild B, Eisler I, Herzog W. Effects of interventions involving the family in the treatment of adult patients with chronic physical diseases: a meta-analysis. Psychother Psychosom. 2010;79(3): 136–148. 10.1159/000286958 20185970

[51] Wright LM, Bell JM. Beliefs and illness: A model for healing Calgary, Alberta, Canada: 4th Floor Press; 2009.

[52] Sebern MD, Woda A. Shared care dyadic intervention: outcome patterns for heart failure care partners. West J Nurs Res. 2012;34(3): 289–316. 10.1177/0193945911399088 21383082

[53] Benzein EG, Hagberg M, Saveman BI. ‘Being appropriately unusual’: a challenge for nurses in health-promoting conversations with families. Nurs Inq. 2008;15(2): 106–115. 10.1111/j.1440-1800.2008.00401.x 18476853

[54] Benzein E, Hagberg M, Saveman BI, Syren, S. Familjefokuserad omvårdnad - ett strategidokument [In Swedish: Family-focused nursing - A strategy document] (Dnr 2010/1733). Kalmar: Centre for Family Research, Linnaeus University; 2010.

[55] Benzein E, Saveman BI. Health-promoting conversations about hope and suffering with couples in palliative care. Int J Palliat Nurs. 2008;14(9): 439–445. 19060795 10.12968/ijpn.2008.14.9.31124

[56] Duhamel F, Dupuis F, Reidy M, Nadon N. A qualitative evaluation of a family nursing intervention. Clin Nurse Spec. 2007;21(1): 43–49. 17213739 10.1097/00002800-200701000-00009

[57] Voltelen B. Family Systems Nursing Intervention: Nurses' experiences and its impact on heart failure families' readjustment processes. A qualitative process evaluation PhD thesis. The University of Southern Denmark. 2016. Available: http://www.forskningsdatabasen.dk/da/catalog/citation?id=2292415073.

[58] Östlund U, Persson C. Examining Family Responses to Family Systems Nursing Interventions: An integrative Review. J Fam Nurs. 2014;20(3): 259–286. 25026964 10.1177/1074840714542962

[59] Benzein EG, Hagberg M, Saveman BI. Att möta familjer inom vård och omsorg [In Swedish: To meet families in nursing and care]. Lund: Studentlitteratur; 2012.

[60] Kitko LA, Hupcey JE, Pinto C, Palese M. Patient and Caregiver Incongruence in Advanced Heart Failure. Clin Nurs Res. 2014 3 5. [Epub ahead of print] 10.1177/1054773814523777 PMC4156554 24599063

[61] Retrum JH, Nowels CT, Bekelman DB. Patient and caregiver congruence: the importance of dyads in heart failure care. J Cardiovasc Nurs. 2012;28(2): 129–136. 10.1097/JCN.0b013e3182435f27 22343213

[62] Hooker SA, Grigsby M, Riegel B, Bekelman DB. The impact of relationship quality on health-related outcomes in heart failure patients and informal family caregivers: an integrative review. J Cardiovasc Nurs. 2015;30(4S): 52–63. 10.1097/JCN.0000000000000270 25955196

[63] SFS, Svensk författningssamling 2001:453 [In Swedish: Swedish Code of Statutes]. Socialtjänstlag [In Swedish: Social Services Act]. Stockholm: Socialdepartementet [In Swedish: Ministry of Social Affairs]. Available: https://www.riksdagen.se/sv/Dokument-Lagar/Lagar/Svenskforfattningssamling/Socialtjanstlag-2001453_sfs-2001-453/#K2

[64] SFS, Svensk författningssamling 1982:763 [In Swedish: Swedish Code of Statutes]. Hälso- och sjukvårdslag [In Swedish: Health Care Act]. Stockholm: Socialdepartementet [In Swedish: Ministry of Social Affairs]. Available: https://www.riksdagen.se/sv/Dokument-Lagar/Lagar/Svenskforfattningssamling/Halso--och-sjukvardslag-1982_sfs-1982-763/

